# The Future of Concurrent Automated Coronary Artery Calcium Scoring on Screening Low-Dose Computed Tomography

**DOI:** 10.7759/cureus.8574

**Published:** 2020-06-12

**Authors:** Jeffrey Waltz, Madison Kocher, Jacob Kahn, McKenzie Dirr, Jeremy R Burt

**Affiliations:** 1 Diagnostic Radiology, Medical University of South Carolina, Charleston, USA; 2 Radiology, Medical University of South Carolina, Charleston, USA; 3 Cardiothoracic Imaging, Medical University of South Carolina, Charleston, USA

**Keywords:** low dose chest ct screening, coronary artery calcium, lung cancer screening, cardiovascular disease, artificial intelligence, deep learning, agatston score, categorical

## Abstract

Low-dose computed tomography (LDCT) has been extensively validated for lung cancer screening in selected patient populations. Additionally, the use of gated cardiac CT to assess coronary artery calcium (CAC) burden has been validated to determine a patient's risk for major cardiovascular adverse events. This is typically performed by calculating an Agatston score based on density and overall burden of calcified plaque within the coronary arteries. Patients that qualify for LDCT for lung cancer screening commonly share major risk factors for coronary artery disease and would frequently benefit from an additional gated cardiac CT for the assessment of CAC. Given the widespread use of LDCT for lung cancer screening, we evaluated current literature regarding the use of non-gated chest CT, specifically LDCT, for the detection and grading of coronary artery calcifications. Additionally, given the evolving and increasing use of artificial intelligence (AI) in the interpretation of radiologic studies, current literature for the use of AI in CAC assessment was reviewed.

We reviewed primary scientific literature dating up to April 2020 using Pubmed and Google Scholar, with the search terms low dose CT, lung cancer screening, coronary artery calcium, EKG/cardiac gated CT, deep learning, machine learning, and AI. These publications were then independently evaluated by each member of our team. Overall, there was a consensus within these papers that LDCT for lung cancer screening plays a role in the evaluation of CAC. Most studies note the inherent problems with the evaluation of the density of coronary calcifications on LDCT to give an accurate numeric calcium or Agatston score. The current method of evaluating CAC on LDCT involves using a qualitative categorical system (none, mild, moderate, or severe). When performed by cardiac imaging experts, this method broadly correlates with traditional CAC score groups (0, 1 to 100, 101 to 400, and > 400). Furthermore, given the high sensitivity of a properly protocolled LDCT for coronary calcium, a negative study for CAC precludes the need for a dedicated gated CT assessment. However, qualitative methods are not as accurate or reproducible when performed by general radiologists. The implementation of AI in the LDCT screening process has the potential to give a quantifiable and reproducible numeric value to the calcium score, based on whole heart volume scoring of calcium. This more closely aligns with the Agatston score and serves as a better guide for treatment and risk assessment using current guidelines.

We conclude that CAC should be assessed on all LDCT performed for lung cancer screening and that a qualitative categorical scoring system should be provided in the impression for each patient. Early studies involving AI for the assessment of CAC are promising, but more extensive studies are needed before a final recommendation for its use can be given. The implementation of an accurate, automated AI CAC assessment tool would improve radiologist compliance and ease of overall workflow. Ultimately, the potential end result would be improved turnaround time, better patient outcomes, and reduced healthcare costs by maximizing preventative care in this high-risk population.

## Introduction and background

Lung cancer (LC) and atherosclerotic cardiovascular disease (CVD) are among the leading causes of death in the United States, with LC accounting for nearly one-quarter of all cancer deaths and CVD being the leading cause of overall mortality in adults [1-2]. Computed tomography (CT) is a well-established screening tool for both lung cancer and coronary artery atherosclerosis detection in addition to a predictor of subsequent CVD adverse outcomes. Low-dose CT (LDCT) is widely used as the test of choice in lung cancer screening programs as a result of multiple, large, multicenter trials demonstrating a reduction in mortality and subsequent Grade B recommendation by the US Preventative Services Task Force (USPSTF) for high-risk individuals [3-6]. This government-backed effort is further supported by the American Cancer Society and the American College of Radiology [7]. The use of LDCT for LC screening has also been investigated by multiple European studies, the largest of which was the Nederlands-Leuvens Longkanker Screenings Onderzoek (NELSON) trial, which also supported the use of LDCT in the screening of LC in the appropriate population [8].

According to the 2015 National Health Interview Survey, over eight million people meet the USPSTF criteria for lung cancer screening, with only 4.4% of them receiving appropriate LDCT screening [9]. While these patients are at an increased risk of lung cancer because of their history of tobacco use, it is also well-established that they are also at risk for the development of atherosclerotic CVD [10-11].

It is well-documented that CAC scoring is a predictor of CVD risk. A prior study by Shemesh et al. demonstrated that a CAC score of 4 or greater is a significant predictor of cardiovascular death (odds ratio 4.7, P < .0001) [12]. The use of CAC as a marker for the presence of coronary artery atherosclerosis has traditionally been quantified with dedicated cardiac CT using an electrocardiogram (ECG)-gated technique [13-14]. Emerging evidence suggests that CAC can be accurately assessed on non-ECG gated CT as well as LDCT. In addition, an increase in CAC seen on LDCT has been associated with an increased risk of negative cardiovascular events [15]. Naturally, it has been hypothesized that CAC could be concurrently acquired on LDCT lung cancer screening, ultimately obtaining two risk assessments on a single exam. The prospect of reducing radiation dose, preventing additional costly tests, and avoiding unnecessary procedures has motivated this effort.

Artificial intelligence (AI) is a rapidly developing technology integrating into the medical field with a particular interest in the interpretation of radiologic exams. Automated AI software for detecting and quantifying CAC has been developed and challenged, with both promising results and limitations exposed. In this comprehensive review, the use of LDCT for concurrent lung cancer and coronary artery atherosclerosis screening is discussed, as well as the potential contribution of AI in CAC quantification.

## Review

LDCT and current application

Lung cancer remains to be one of the most common causes of cancer death, with one-quarter of all cancer deaths attributed to it, more than breast, prostate, colorectal, and brain cancers combined [1,7]. Although the incidence is declining, five-year survival is particularly poor (19%) likely due to 57% of patients having metastatic disease at presentation [1]. These statistics have been improving in recent years, with death rates continuing to drop for both men and women, largely due to tobacco smoking cessation in addition to improved screening measures such as low-dose computed tomography (LDCT) [7].

The National Lung Screening Trial (NLST) is the largest trial to date that studied current and former smokers with a greater than or equal to 30 pack-year history and demonstrated a 20% decrease in lung cancer mortality over a three-year follow-up period when screened with LDCT as compared with chest radiography [3]. In addition, the Multicentric Italian Lung Detection (MILD) trial reported a 39% reduction in lung cancer mortality compared with no intervention in patients with a greater than or equal to a 20 pack-year history and after longer follow-up [4]. Further work by the International Early Lung Cancer Action Program (IELCAP) demonstrated a clear mortality benefit in patients treated for stage I cancer detected on CT as compared to those who were not treated, with the clinical implication that CT screening has the potential to detect lung cancer that is curable [5].

In terms of implementation cost, Black et al. estimated that the NLST screening measures equated to less than $100,000 per quality-adjusted life-year gained [16]. All of these previous studies, in addition to the demonstration of the cost-effectiveness of LDCT screening, led to its Grade B recommendation by the US Preventative Services Task Force (USPSTF) in 2014 for adults aged 55 to 80 years old who have a 30 pack-year history and currently smoke or have quit within the past 15 years [6]. With this recommendation, private insurances are mandated to cover the cost of screening. The American Cancer Society currently recommends annual lung cancer screening for patients aged 55 to 74 years who are current or former heavy smokers and are in relatively good health [7]. In summary, LDCT is a clearly proven screening tool that has widespread acceptance and mortality benefits in a subset of patients.

Coronary artery calcium scoring and its prognostic value

Atherosclerotic cardiovascular disease is the overall leading cause of death in the United States, manifesting as coronary heart disease (CHD), ischemic heart disease, and ischemic stroke [2]. Alarmingly, up to half of all coronary deaths are not preceded by cardiovascular symptoms [17]. The development and post-diagnostic course of CHD has a long asymptomatic period, which, theoretically, should afford robust and thorough screening measures [13].

**Figure 1:**
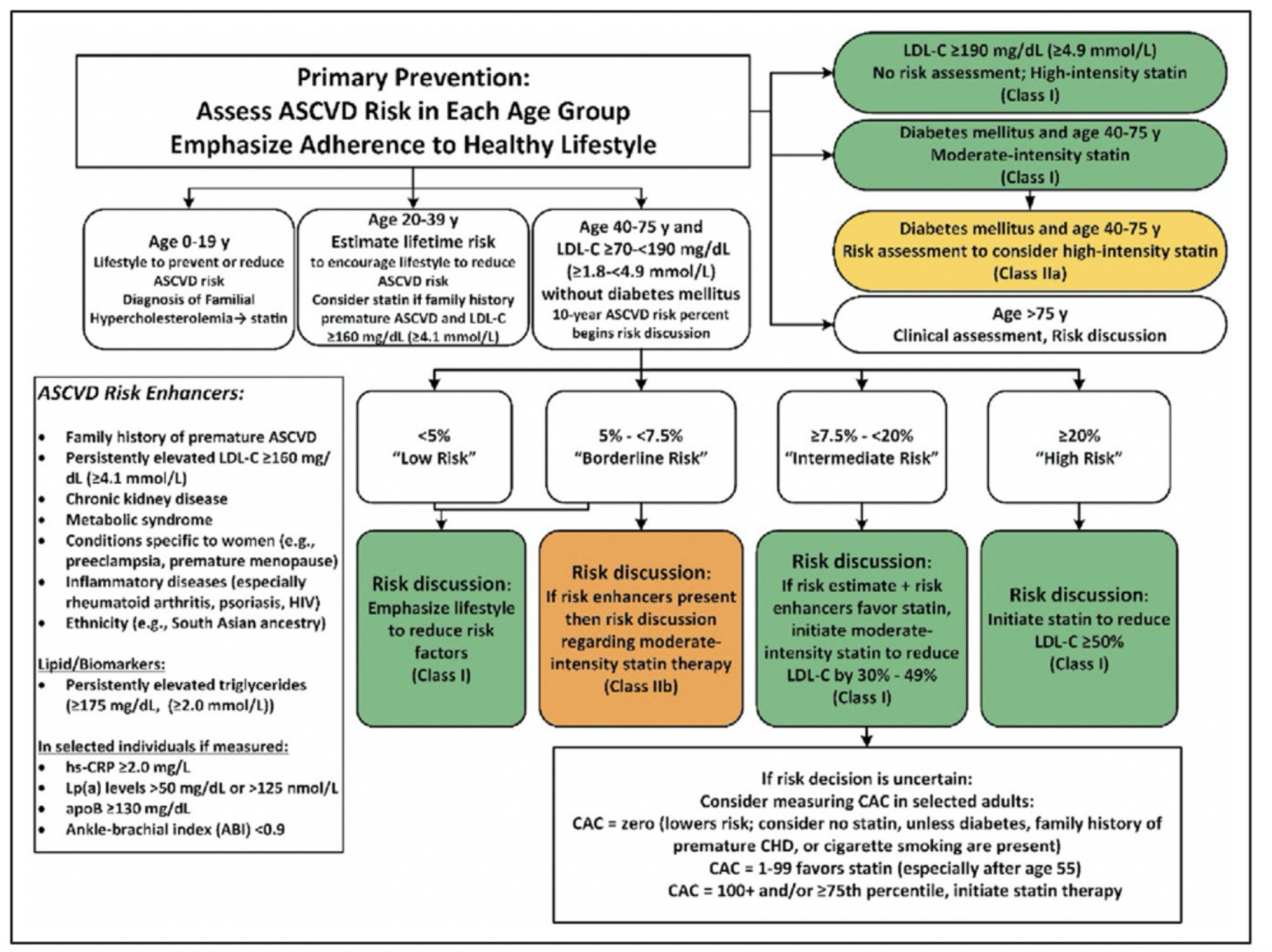
American Heart Association and American College of Cardiology guidelines for primary prevention of atherosclerotic cardiovascular disease (ASCVD) Determination of CAC is included for patients at “intermediate risk.” ABI: ankle-brachial index; apoB: apolipoprotein B; ASCVD: atherosclerotic cardiovascular disease; CAC: coronary artery calcium; CHD: coronary heart disease; HIV: human immunodeficiency virus; hs-CRP: high-sensitivity C-reactive protein; LDL-C: low- density lipoprotein cholesterol; Lp(a): lipoprotein (a) Reproduced with permission from Grundy et al. [18], copyright © 2018, American Heart Association, Inc. and American College of Cardiology Foundation.

Although there are many multivariate screening measures for risk assessment, including the presence of diabetes, smoking, hypertension, cholesterol, and age, these predictive scores lack the ability to measure actual severity at the time of assessment, in addition to overall risk stratification. All of these factors also have variable predictive sensitivity for overall CHD mortality. The American Heart Association has published proposed guidelines for the use of β-Hydroxy β-methylglutaryl-CoA, also known as 3-hydroxy-3-methylglutaryl-CoA (HMG-CoA) reductase inhibitors (statins) and next-step treatments for atherosclerotic cardiovascular disease (ASCVD) based on lifestyle, risk assessment, age, and blood cholesterol levels (Figure 1) [18]. While helpful in general treatment guidelines, they lack a way to adequately assess the present disease burden.

Coronary artery calcification can be used as a surrogate measure when evaluating for atherosclerotic coronary artery plaque burden, and multiple population-based cohort studies demonstrate CAC measurements to be strongly predictive of future cardiovascular events [19]. The two primary methods for quantifying CAC is through objective measurement of the density of coronary artery calcifications, called Agatston scoring, and by CAC volume, which is a 3D assessment of the CAC burden. The Multi-Ethnic Study of Atherosclerosis (MESA) trial studied a cohort of 6,814 patients of four different ethnicities and demonstrated that CAC outperformed traditional risk models in the prediction of cardiovascular events for each of the ethnicity groups [20-21]. The hazard ratio (HR) for any coronary event based on the CAC score was 3.1 for a CAC score of 1-100, 7.73 for a CAC score of 101-200, and 9.67 for a CAC score of 201-300, all with a p-value <0.0001 [21]. Similar results were found in several other population-based studies such as the Heinz Nixdorf Recall (HNR) study, the Prospective Multicenter Imaging Study for Evaluation of Chest Pain (PROMISE) study, and the Rotterdam study [22-24]. The Framingham study added additional information to the assessment of the Agatston score, including CAC distribution and coronary dominance, showing synergistic significance in predicting subsequent CHD [25].

It has been shown that the total amount of CAC provides superior prognostic information about the risk of future CHD, especially when compared to traditional risk factor assessment [13,26]. Patients with high CAC scores greater than 100 to 300 have been observed to have a high rate of subsequent CHD events, making them a high yield patient subset for intervention [13]. This predictive value has proved to be even stronger in patients with diabetes, where the CAC score was shown to be a highly significant independent predictor of CHD events [27]. While CAC is commonly assessed using cardiac CT, there is much to be studied regarding its potential benefit when obtained from noncardiac chest CTs.

Current standard of care for CAC analysis

The current method for CAC scoring is the acquisition of a 120 kVp non-contrast-enhanced, prospective ECG-triggered cardiac CT [13-14]. Images obtained can then be used to evaluate the extent of calcification and assign a quantity-based total called the Agatston score determined by lesion area in square millimeters and the maximal CT Hounsfield unit [13,28]. The benefits of using coronary artery calcification CT include the detection of CAC in patients before they exhibit symptoms allowing for improved risk stratification and appropriate preemptive management. Additionally, this method for CAC scoring is already commonly implemented in routine practice and there is good familiarity with it among general radiologists, cardiologists, and primary care practitioners [26].

However, this form of imaging also presents drawbacks. In a study done by Kim et al., multi-detector CT (MDCT) performed to measure CAC severity was found to expose patients to varying amounts of radiation, potentially putting them at increased risk for radiation-related cancers [26]. Given these concerns with long-term safety, the appropriateness of screening asymptomatic patients must consider the age of the patient [13]. Thus, cardiac CT is not advised for younger patients (generally, males under 40 years of age and women under 50 years of age) in whom the risks of exposure do not outweigh the expected benefits associated with early detection in an older, high-risk population [13].

Current published guidelines primarily suggest limiting CAC testing to asymptomatic patients with low to intermediate risk of CHD. The 2010 ACC/AHA guidelines assigned CAC scoring a Class IIA recommendation for intermediate-risk patients and a Class IIB recommendation in low- to intermediate-risk patients [13]. Additionally, per the 2019 ACC/AHA guidelines for primary risk prevention, it is reasonable for patients who are at intermediate risk (7.5% to <20% 10-year ASCVD risk) or selected borderline-risk (5% to <7.5% 10-year ASCVD risk) to have additional workup (class IIA recommendation) such as CAC score for the purpose of treatment optimization [29]. Most guidelines suggest using CAC scoring for shared decision-making in cardiovascular disease workup and stratification (Figure 1) [30-32]. Currently, the 2018 USPSTF cites insufficient evidence of the addition of CAC scoring to traditional risk assessment for the prevention of cardiovascular disease.

The 2013 ACC/AHA guidelines address the recognition of CAC as an identifiable variable when evaluating cardiovascular disease [30]. These guidelines designate CAC as a Class IIB recommendation. The main use for CACS described by the authors is to help guide patient management plans. CAC scores can be used to better assess a patient’s risk of CVD and assign a more appropriate risk category. Subsequent treatment paradigms can then be chosen, as opposed to treatment dictated solely based on lipid profile [30]. For example, patients with borderline to intermediate risk profiles with an Agatston score of 100 or greater will have a risk of cardiovascular events greater than 7.5% in 10 years and thus would have a risk-benefit ratio favoring the use of higher-dose statins and possibly acetylsalicylic acid (ASA) [29]. This is in contradistinction to a person with borderline to intermediate risk and a CAC score of 0, which would have a cardiovascular event risk of <7.5% in 10 years and may not warrant the use of higher-dose statin or ASA. The additional information in these cases allows for better, shared decision-making between providers and patients [29].

LDCT and concurrent coronary artery calcium scoring

**Figure 2:**
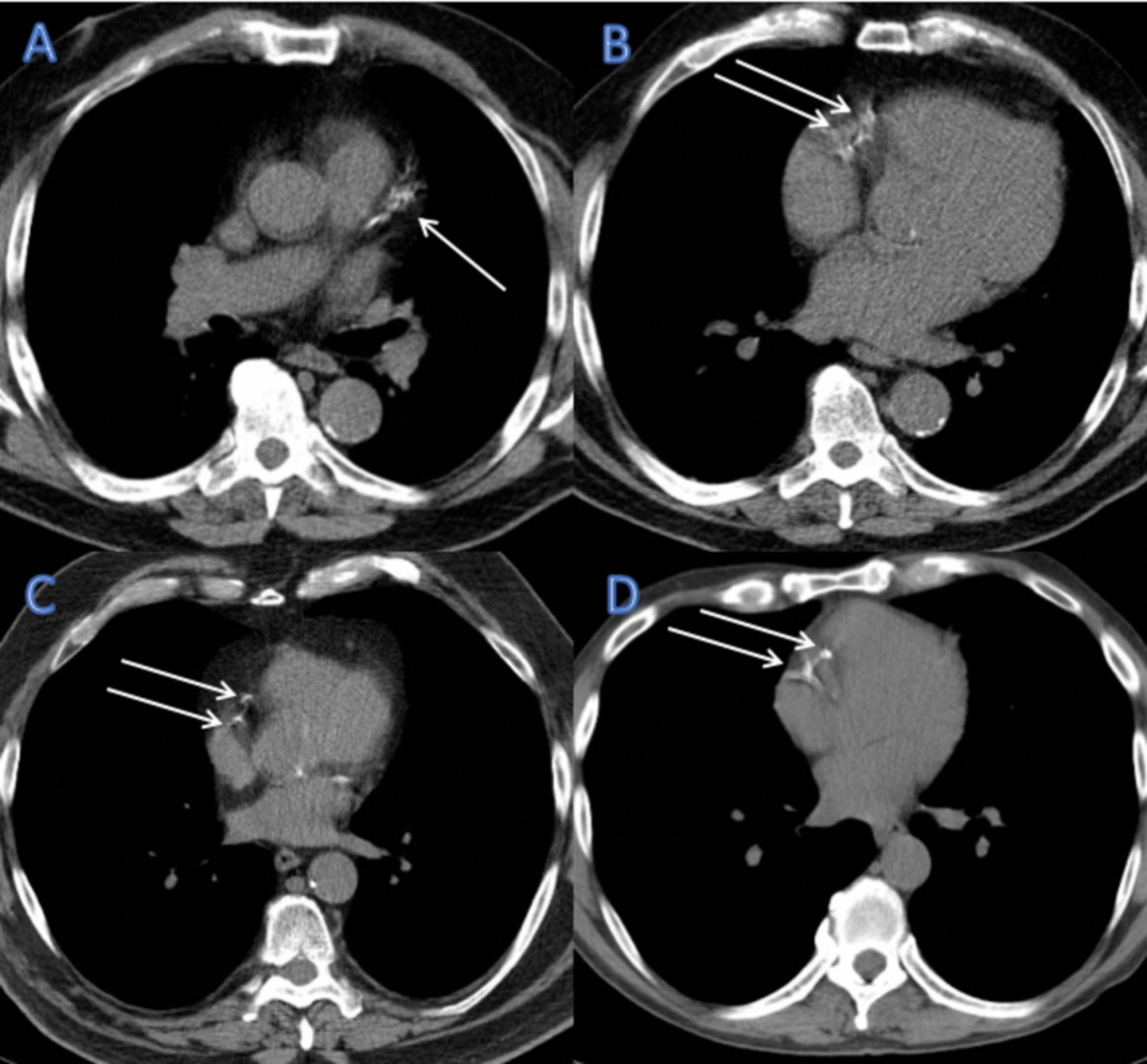
Example of cardiac motion artifact Axial noncontrast-enhanced, non-ECG gated chest CT images performed at our institution for lung cancer screening demonstrating the effect of cardiac motion artifact (arrows). Left anterior descending artery motion is characterized by a smudged appearance (A). The right coronary artery is the most commonly affected epicardial artery (B, C, and D) with artifactual duplication of the vessel. ECG: electrocardiogram; CT: computed tomography

There is a significant overlap in the patient populations for whom lung cancer screening is indicated and for which CAC scoring has been shown to be of benefit. A screening program that uses a single imaging acquisition could potentially optimize risk assessment with a lower overall radiation dose and a resultant reduction in healthcare costs. Although CAC scoring is generally performed on ECG-synchronized cardiac CT (GCT), it can also be measured on any standard protocol CT image that includes complete imaging of the heart such as routine noncontrast-enhanced, non-ECG gated chest CT (NCT) scans. There are several inherent problems with utilizing NCT, including cardiac motion, which could potentially limit the accurate assessment of CAC (Figure 2). Several studies have compared the CAC score measured on NCT with concurrent GCT and demonstrated good general agreement. For example, Budoff et al. demonstrated intraclass correlation coefficients (ICC) of 0.96 and 0.97 for Agatston and volume scoring, respectively (p=0.0001) [23]. Chen Hu et al. measured 94.8% sensitivity and 100% specificity for determining positive CAC, defined as CAC >0, on NCT with slightly better ICC [33].

Several studies have also looked specifically into the feasibility of using LDCT, for which the patient would presumably already be receiving for lung cancer screening, for a concurrent assessment of CAC. Wu et al. found that the binary identification of CAC on LDCT as present or absent was highly concordant with regular-dose ECG-gated MDCT, indicating that LDCT is very sensitive for CAC and that the absence of CAC on LDCT precludes the need for additional CT imaging of the heart [34]. They also determined that when performed with an optimized protocol, LDCT is reliable for the categorization of CAC into the four major Agatston score ranks [34]. Kim et al. also found an excellent degree of reliability in the visual ranking of CAC on LDCT as compared to scoring on cardiac gated CT, indicating that a reliable categorical ranking of CAC by Agaston score can be made on LDCT [35].

However, the viability of using a low-dose chest CT, like those used for lung cancer screening, to assess CAC and specifically for the assessment of Agatston calcium scoring has been shown to be less accurate. Even on standard protocol chest CT studies, Budoff et al. noted an overestimation of Agatston score on NCT with increasing Agatston scores on GCT [23]. Deprez et al. investigated the use of lower kilovolt potential during scanning to decrease the patient dose and found an overestimation of the Agatston score when compared with standard GCT techniques [36]. This change in the Agatston score is related to the known alteration in the density of the calcium with changes in kV. An alternative protocol to dose reduction without changing the density of the calcium would be a reduction in the tube current while maintaining the standard kV of 120 with iterative reconstruction or the use of mass/volume calcium scoring, which is less dependent on tube potential [36]. Investigations into the optimal slice thickness and Hounsfield units (HU) for detecting CAC have also been performed, with a recommendation of 2.5 mm thickness and a 130 HU threshold for maximum accuracy [37].

While the variability of Agatston scores on LDCT and cardiac CT is high, Ming-Ting Wu et al. found that CAC scores categorized into four score ranks, based on Agatston units, is highly concordant between the studies (κ = 0.89 for both observers). Therefore, while measuring the Agatston score on LDCT is not highly accurate, the determination of overall calcium volume is reliable for risk stratification [34]. While a formal Agatston score may not be currently obtainable from non-gated LDCT, CAC volume scores are accurate for quantification [23]. When CAC volume scoring is not available, qualitative CAC assessment performed by cardiac imaging experts (none, mild, moderate, or severe) also correlates with Agatston score groups (0, 1 to 100, 101 to 400, and >400) [38]. However, qualitative methods are not as accurate or reproducible when performed by general radiologists [35]. Ultimately, new categorization thresholds would need to be explored and defined for optimal risk assessment based on non-gated LDCT [39].

Not only does CAC scoring allow for risk stratification in terms of future cardiovascular events, but when used in conjunction with LDCT lung findings, it can be used to predict the overall mortality of lung cancer patients. In a previous study, Yan et al. selected 180 subjects from the National Lung Screening Trial (NLST) and calculated Agatston scores for their initial LDCT. Then, a deep learning technique was developed using both chest LDCT images and Agatston scores as input factors to predict all-cause lung cancer mortality, which demonstrated superior performance when compared to either factor alone [40]. The predictive value of LDCT clearly has unrealized potential.

The role of artificial intelligence in quantifying CAC

**Figure 3:**
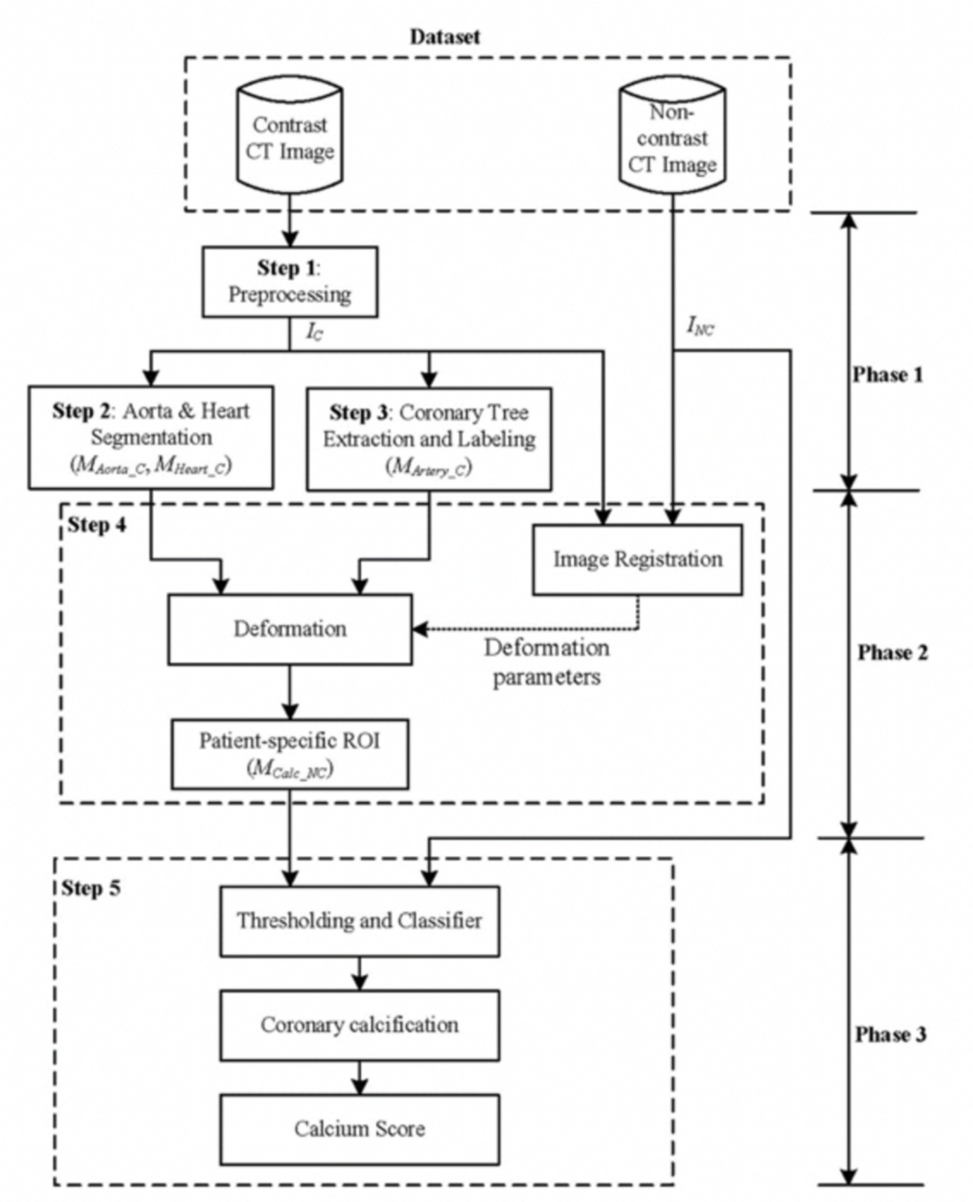
Example of an artificial neural network framework used to automatically detect the coronary artery calcium score from non-contrast chest CT IC (contrast image after preprocessing), INC (noncontrast image), MAorta_C, MHeart_C, MArtery_C (segmentation results of the aorta, heart, and coronary arteries in the contrasted image), MCalc_NC (the patient-specific ROI for calcification detection defined on the noncontrast image) Reproduced with permission from Sandstedt et al [41], copyright © 2019, European Radiology http://creativecommons.org/licenses/by/4.0/

CAC scoring has been traditionally measured by imaging specialists with a semi-automatic software, starting with the manual demarcation of the coronary artery calcifications and subsequent computer calculation of the Agatston score. With greater volumes of studies being performed and the potential of CAC scoring being added to other imaging protocols, there is a growing need for a more efficient and automated method [41]. The primary problem with the development of an automated system arises from the fact that calcifications are not solely present in the coronary arteries but also in surrounding cardiac tissues and cardiac valves, making automated detection complicated [42]. Several prior studies have attempted to solve this problem first by designating a region of interest around the heart on cardiac CT images and then identifying relevant CAC within the region by combinations of their relative position, texture, or size [43-46]. While this requires labor-intensive coronary artery and cardiac segmentation, more recent studies have used deep-learning algorithms. Yang et al. proposed an algorithm on producing an automatic method for calcium scoring by using data from GCTs to create segmentation maps, which were then applied to the NCT datasets (Figure 3).

**Figure 4:**
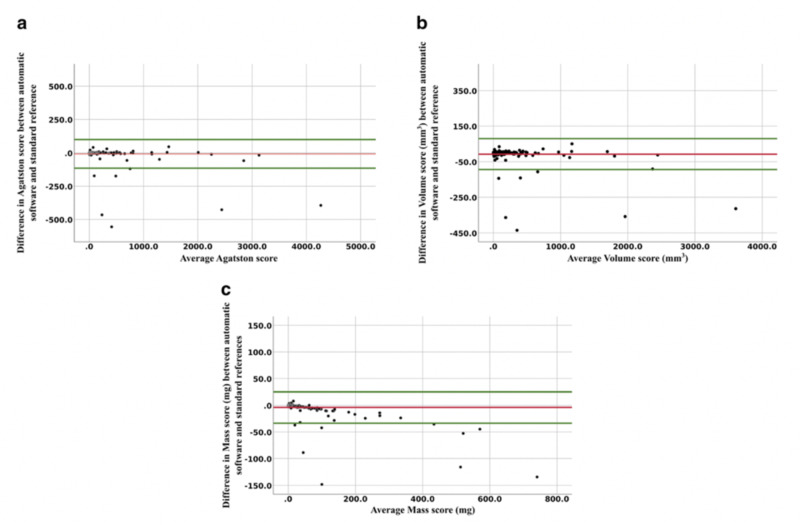
Bland Altman analyses showing the difference in coronary calcium score between AI software and standard reference, plotted against the mean of the coronary calcium measurements The red line represents mean differences with the upper and lower limits of agreement with a 95% confidence interval in green. (a) Agatston score mean difference of -8.2 (limits of agreement -115 to 98.2), (b) volume score mean difference of -7.2 (limits of agreement -7.2 to 79.1), and (c) mass score mean difference of -3.8 (limits of agreement -33.6 to 25.9). AI: artificial intelligence Reproduced with permission from Yang et al [42], copyright © 2016, Medical Physics, John Wiley and Sons.

The AI boom has substantially expanded the role of automated detection and measurement, with the development of expansive labeled datasets in addition to deep learning algorithms. A recent study by Sandstedt et al. described completely automated software that utilized machine learning trained on annotated routine GCTs. The algorithm was then used for CAC scoring using 315 GCTs and validated against semi-automatic scoring on the same scans [41]. They were able to show that the algorithm had excellent correlation and agreement with the semi-automatic manual method but took less time (Figure 4). Another study developed a deep learning regression framework to estimate the Agatston score on NCT achieving a high Pearson correlation coefficient in relation to the reference standard (p=0.932; p<0.0001), especially in the context of using noisier images with comparatively more motion artifact [47]. This convolutional neural network was developed to not require an annotated image dataset, only using the input values of the NCT and the measured Agatston score for training [47].

AI screening potential for automated CAC in LDCT scans

**Figure 5:**
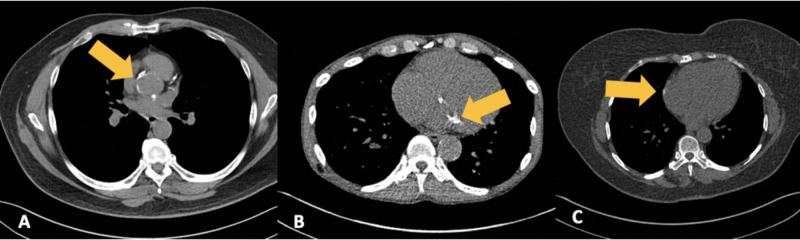
Miscategorized calcifications by AI software LDCT examinations performed at our institution in patients with different examples of calcifications that may be miscategorized as coronary artery calcifications by AI software. (A) Aortic annulus calcifications, (B) mitral annulus calcifications, and (C) pericardial calcifications, all have the potential to be misidentified, and deep learning algorithms must have the ability to account for calcification location. AI: artificial intelligence; LDCT: low-dose computed tomography

With the prevalence and general acceptance of LDCT screening for lung cancer, the added benefit of concurrent CAC measurement and further risk stratification of patients has yet to be fully realized. Although visual scoring of CAC on LDCT scans performed by cardiac imaging experts has been shown to have good concordance when compared to the standard Agatston score and ECG-gated calcium scoring CT, there remains potential for miscategorization and variation in inter-rater reliability, necessitating an automated, objective measurement tool [35,48]. Many prior automatic calcium scoring methods utilized contrast-enhanced cardiac CT images, which allowed for the segmentation of the coronary arteries and subsequent localization of the calcifications [49]. Routine noncontrasted chest CTs and LDCT do not afford the luxury of coronary artery visualization or segmentation; therefore, additional measures are needed to accurately identify, localize, and measure these calcifications. Many different calcifications have the potential to be misidentified as CAC such as the aortic valve, mitral annulus, and pericardial calcifications, particularly on motion-prone LDCT (Figures 5A-5C). One method to combat potential misidentification is to segment aortic and cardiac structures and subsequently localize the calcifications to determine eligibility as CAC [50].

In a study performed by Isgum et al., a novel algorithm was developed to approximate positions of coronary calcifications as inferred by a probabilistic coronary calcium map using data from low-dose, noncontrast-enhanced, non-ECG-gated chest CT scans [44]. Their results indicated an underestimation of CAC, as noise reduction features, as well as spatial features, have a tendency to generate false negatives [39,44]. Further studies are needed to find the right balance of sensitivity and specificity while maintaining cost-effectiveness.

Future studies

Future studies are needed prior to the routine application of automated CAC into LDCT screening. With the potential detection of both early lung cancer and asymptomatic atherosclerotic disease, this may have a large impact on both overall mortality in addition to health value and expenditures. This comes at the risk of unnecessary CHD work-ups, additional costly imaging, and unwarranted treatments or procedures. Further investigative studies are needed to evaluate the costs and benefits, especially in terms of quality-adjusted life years, if such a recommendation is to be implemented.

Although it has been established that LDCT scans can be used to detect CAC and that it is predictive of incident CHD events, there is great variation as to how the CAC should be scored or reported. The inherent nature of routine noncontrast chest CT does not allow for accurate Agatston scoring, therefore, only correlations between the true Agatston score and the noncontrast CT-derived CAC volume can be calculated. Further studies are required to evaluate the predictive value of AI-generated CAC volume from these LDCT scans. Methods of coronary artery segmentation and probabilistic calculations of expected CAC also need to be further evaluated as well as the ideal characterization features to best label them. Threshold values could be gauged according to their actual cardiac event predictive value.

## Conclusions

We systematically reviewed the level of published evidence regarding concurrent screening for CHD and lung cancer using low-dose chest CT. The importance of screening for CHD is based on the high morbidity and mortality of the disease, coupled with the proven effectiveness of secondary prevention. Previous studies have shown that CAC detection is an effective tool to measure the risk of cardiovascular disease in asymptomatic patients. In addition, up to half of coronary deaths occur in patients with no prior coronary symptoms, furthering the importance of CHD screening in at-risk populations. The current standard of care is the determination of risk using clinical risk factors with the option to perform cardiac CT for borderline and intermediate-risk patients. However, there is a great potential benefit in detecting CAC in patients undergoing LDCT for lung cancer screening. Currently, there is a robust system in place for lung cancer screening using LDCT, which is another major cause of mortality. There is a large overlap in patient populations between those at risk for lung cancer and CHD, and early identification in both cases improves outcomes. Furthermore, both can be screened by a review of clinical risk factors and CT of the chest. The feasibility of a single screening tool to assess both diseases would be of great benefit, financially and medically. In our review, we found that many studies show LDCT is a viable option for simultaneous lung cancer and CHD screening. When evaluating CAC on LDCT, it is currently evaluated using a categorical system (none, mild, moderate, or severe). This method correlates with traditional CAC Agatston score groups (0, 1 to 100, 101 to 400, and >400) when performed by a cardiac imaging expert. However, qualitative methods are not as accurate or reproducible when performed by general radiologists. The implementation of AI in the screening process gives a quantifiable and reproducible numeric value to the calcium score and more closely aligns with the Agatston score. Additionally, AI increases the speed of the tedious process of CAC scoring and adds automation to the screening process. Ultimately, the end result would be improved turnaround time, better patient outcomes, and reduced healthcare costs by maximizing preventative care in this high-risk population. Early studies involving AI for the assessment of CAC are promising, but more extensive research involving AI scoring of CAC on LDCT for lung cancer screening is needed.
